# The Mediterranean Diet and Breast Cancer: A Personalised Approach

**DOI:** 10.3390/healthcare7030104

**Published:** 2019-09-09

**Authors:** Amani Al Shaikh, Andrea J. Braakhuis, Karen S. Bishop

**Affiliations:** 1 Discipline of Nutrition and Dietetics, School of Medical Sciences, Faculty of Medical and Health Sciences, University of Auckland, Auckland 1023, New Zealand (A.A.S.) (A.J.B.); 2 Auckland Cancer Society Research Centre, School of Medical Sciences, Faculty of Medical and Health Sciences, University of Auckland, Auckland 1023, New Zealand

**Keywords:** breast cancer, Mediterranean-style dietary pattern, personalised nutrition, nutrigenetics, polyphenols, diet–gene interactions, nutrigenomics, micronutrients

## Abstract

There have been many original and review articles summarizing the impact of nutrition and diet on breast cancer risk. However, very few consider the implication of genetic background and the effect of personalised nutrition on the risk and prognosis of breast cancer. A literature search was performed using the following databases: MEDLINE (Ovid), PubMed, Scopus and EMBASE (Ovid). The ensuing search terms were selected: genomics, nutrigenomics, breast cancer, breast neoplasms, cancer, nutrigenetics, diet–gene interaction, and Mediterranean, nutrition, polyphenols and diet. In this review, we discuss the Mediterranean-style diet and associated nutrients, evidence of benefit, impact on gene expression and evidence of interactions with genotype and how this interaction can modify breast cancer risk and progression. In addition, the impact of nutrients commonly associated with a Mediterranean-style diet, on breast cancer treatment, and synergistic effects are mentioned when modified by genotype. Some evidence exists around the benefit of a gene-based personalised diet based on a Mediterranean-style dietary pattern, but further evidence in the form of clinical trials is required before such an approach can be comprehensively implemented.

## 1. Introduction

The incidence of non-communicable diseases such as cancers, cardiovascular diseases and diabetes has increased with an aging population. However, aging is not the only risk factor for non-communicable diseases. Lack of physical exercise, excess calorie intake and a diet low in unprocessed foods, fruit and vegetables, and high in meat, fats, salt and sugars have contributed to the rise in non-communicable diseases [1]. Based on data from 195 countries (between 1990 and 2017), the leading causes of diet-related death and morbidity were a high intake of salt and a low intake of whole grains and fruit [2]. Clearly, diet can be used to improve health and prevent avoidable deaths.

The excitement surrounding a personalised approach to diet comes from a growing awareness of the potential for modifications of food or diet to reduce the risk of non-communicable diseases. This is as a result of the increasing knowledge of the importance of the individuals’ genetic response to diet [3]. It is an emerging field that has the potential to unfold the impact of nutrition on gene expression, and it brings together the science of bioinformatics, nutrition, epigenetics, genomics, epidemiology, and molecular medicine. 

This review focuses on the effect of the Mediterranean-style diet (MD), and relevant food-related bioactive compounds, on the risk and progression of breast cancer. It encompasses a general overview of nutrigenomics, the role of single nucleotide polymorphisms (SNPs) in diet–gene interactions and how components of the MD may influence epigenetics and thus modify gene expression and impact breast cancer risk.

Breast cancer ranks as the second most frequent type of cancer globally, and the fifth leading cause of cancer-related deaths overall [4]. In women, breast cancer is considered the most commonly diagnosed cancer in the vast majority of countries around the world, making up approximately one-quarter of all cancers diagnosed [4]. The high incidence of breast cancer can be attributed to a variety of factors, most importantly increasing age and lifestyle. Migration to urban areas is also considered a palpable factor associated with increased incidence [5,6,7] due to the lifestyle change this migration gives rise to. This increase in incidence is largely attributed to the adoption of a Westernised lifestyle, whereupon unhealthy dietary habits are adopted and physical activity is decreased [5,6,7]. The effect on breast cancer incidence is more evident amongst premenopausal than postmenopausal women [8]. A Western diet is typically low in fresh fruit and vegetable intake and high in animal fats, processed foods, salt and sugars.

Diet is an important pillar of any lifestyle, and can be used as a beneficial factor to help prevent cancer in general, and breast cancer in particular [9]. In addition, it may also reduce the risk of cancer progression [9] and thus improve treatment outcomes and decrease human suffering. We recognise that a reduction in breast cancer risk requires a holistic approach but in order to obtain good-quality scientific evidence, we need to address the various components individually. For this reason, we consider the interaction between numerous foods/nutrients and genotype/gene expression, and their influence or potential influence on breast cancer.

Genetic variants are relevant not only to breast cancer risk, but genotype may influence an individual’s nutritional behaviour as well as response to specific nutrients. For example, some genetic variations related to adiposity could affect energy intake by influencing satiety/appetite [10]. In addition, personal preference for specific foods, such as sugar and carbohydrate, is identified as an effect of genetic variation [11,12], with sugar consumption being explained by 48% of the genetic variation [11]. Furthermore, in some cases, there are interactions between dietary patterns/nutrients and genotype, and this interaction could impede or accelerate breast cancer risk and/or progression [13,14,15,16,17]. The genotype (CC vs. CT/TT) of the catalase gene, which helps determine the functioning of the catalase antioxidant enzyme, may also influence the benefit received from consuming adequate quantities of fruit and vegetables [13]. The CC genotype resulted in a 17% reduced risk of breast cancer development [13].

Breast cancer is a disease that may develop and/or progress due to several possible reasons, including non-modifiable factors such as increasing age and genotype, as well as modifiable factors such as smoking, alcohol consumption, lack of physical activity, poor diet and obesity. Obesity and being over-weight play a critical role in increasing the risk of breast cancer recurrence and arises due to an energy imbalance. In a meta-analysis of 43 studies carried out by Rock et al., the risk of recurrence was markedly higher amongst obese breast cancer survivors than those who were not obese [18]. A recent study revealed an interaction between diet–gene predisposition and long-term changes in body mass index (BMI) and body weight in two independent prospective cohorts of US men and women [19]. 

In addition to an energy imbalance, the interaction between several dietary nutrients and gene expression is of interest in the field of tumorigenesis. For example, the protein levels of the tumour suppressor gene, *p*53, were decreased due to a high intake of linoleic acid (commonly found in plant oils) [20,21]. However, the expression of *p53* was up-regulated by docosahexaenoic acid (DHA) [20,21], which is commonly found in oily fish. Another example of such an interaction is demonstrated by dietary folate (commonly found in dark green vegetables and legumes), the consumption of which may influence the hyper-methylation status of gene promoters on the *retinoic acid receptor beta* (*RARB*) or *breast cancer one* (*BRCA1*) genes. This interaction was demonstrated amongst 146 Iranian women with breast cancer in an age-dependent manner [22]. The aforementioned studies illustrated the notable impact of dietary intake on gene expression, and therefore a potential impact on breast cancer incidence and recurrence.

The MD diet is one of the most popular dietary patterns currently being examined in the field of cancer prevention and treatment outcomes. The Mediterranean-style dietary pattern is a dietary style followed by populations living around the Mediterranean Sea [23]. The dietary pattern is based on the consumption of unrefined cereals, fresh fruit and vegetables (leeks, mallow, tomatoes, lettuce, chicory, broccoli, and mushrooms, amongst others), fresh water, wine and olive products, and supplemented by sheep cheese, very little red meat intake and a strong propensity for fish and seafood [19]. However, there are variations in the diet based on culture and locally available produce [23], and hence we have described it as a dietary pattern. Van den Brandt and Schulpen investigated the impact of adherence to a Mediterranean-style diet in over 2000 cases of breast cancer in the Netherlands, and found that adherence to the dietary pattern in estrogen receptor negative (ER-) and ER-/progesterone receptor negative (PR-) postmenopausal breast cancer decreased incident risk [24] These results were confirmed by a meta-analysis. In this analysis, they excluded alcohol [24], as there is a known increased risk between alcohol consumption and breast cancer.

**Figure 1:**
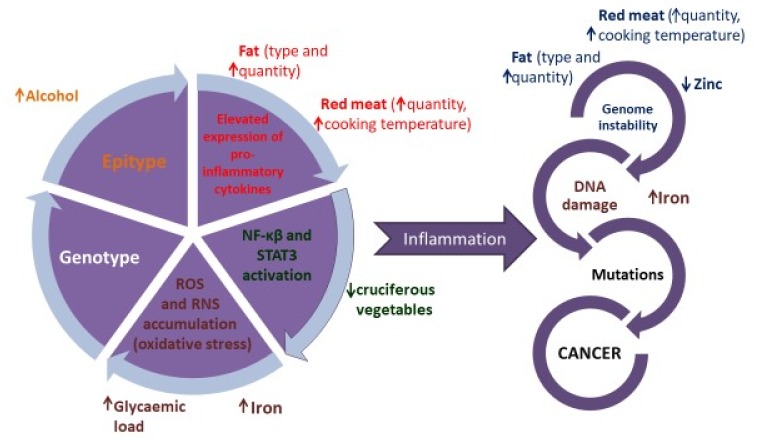
The influence of diet on the series of events that mediate inflammation-induced cancer initiation. The colour of the text links the nutrient/food to the mechanism of action. = high intake can be harmful and = low intake can be harmful. Abbreviations: NF-κβ—nuclear factor kappa-light-chain-enhancer of activated B cells; RNS—reactive nitrogen species; ROS—reactive oxygen species; STAT3 - Signal Transducers and Activators of Transcription 3.

We are well aware that high alcohol consumption is associated with harmful effects on health, some of which include the increased incidence of cancers (Figure 1), liver damage and cognitive impairment. However, alcohol toxicity impacts one-carbon metabolism, which is exacerbated by folate deficiency (folate is commonly found in green leafy vegetables). Reactive Oxygen Species (ROS) generated during the metabolism of ethanol can induce deleterious epigenetic changes that can promote the incidence of cancers by reducing the expression of tumour suppressor genes. These epigenetic changes include methylation of gene promoter regions (of tumour suppressor genes) and histone modifications. Although not specific to breast cancer, we know that epigenetic modifications can impact cancer risk, and that certain dietary patterns/foods/nutrients can modify the epigenome [25]. 

Interestingly, implementing a healthy dietary intervention could not only decrease incidence, but also indirectly improve outcome in breast cancer survivors by promoting weight loss and reducing obesity [26,27,28,29,30,31,32]. In this context, following a dietary pattern that consists of the basic principles of the Mediterranean-style diet is reported in several nutritional studies to be linked with better health outcomes [33] and has been suggested as a standard of nutritional quality due to its main components [34]. However, to date, there is insufficient evidence to support personalised (gene-based) nutrition for the treatment of obesity [35].

This review explores evidence to convey that the Mediterranean-style diet might be a beneficial dietary pattern to influence breast cancer outcomes, as well as the influence of the diet on biomarkers associated with the risk of recurrence. In addition, evidence of the beneficial role of nutrigenomics has been emphasized in terms of breast cancer outcomes in this review. 

## 2. Materials and Methods

A literature search of the interaction between diet, genetic influence and breast cancer was conducted by using various databases, namely MEDLINE (Ovid), PubMed, Scopus and EMBASE (Ovid). The following search terms were selected: genomics, nutrigenomics, breast cancer, breast neoplasms, cancer, nutrigenetics, diet–gene interaction, and Mediterranean, nutrition, polyphenols and diet. We also applied pearl growing to identify other suitable citations. Our strategy employed the use of medical subject headings (MeSH terms) where possible, but in circumstances when a MeSH term was invalid in a particular database, an equivalent term was substituted. Other than nutrigenomics we have also reviewed some of the works performed in the areas of diet supplementation, diet–gene interaction and dietary guidelines.

Publications were selected if they included genotype or gene expression testing and diet-related outcome or intervention in breast cancer risk and/or progression. Studies on non-breast cancer patients, or patients not at risk of breast cancer, were included if deemed relevant to the discussion. 

## 3. Mediterranean Diet and Breast Cancer

Diet is considered one of the most important modifiable factors contributing to cancer prevention [9]. Adherence to the Mediterranean-style dietary pattern has been suggested as a beneficial factor for preventing breast cancer [29]. In addition, close adherence to the Mediterranean-style dietary pattern has shown an improvement in breast cancer survivors’ general health by decreasing their risk of non-breast cancer-related mortality [31]. Castello et al. found that the Mediterranean-style dietary pattern showed a protective role against breast cancer mortality risk, particularly in the case of triple-negative breast cancer [8]. The link between dietary intake and breast cancer might be attributed to the indirect effect of specific nutrients on breast cancer due to their influence on inflammation, DNA damage and repair, oxidative stress, and genetic modifications. Herein, we will highlight the effect of nutrients commonly found in a Mediterranean-style diet on breast cancer. These nutrients include fatty acids, polyphenols (epigallocatechin-3-gallate (EGCG), resveratrol, organosulfur compounds, quercetin, kaempferol and apigenin) and micronutrients (zinc and selenium) commonly found in a Mediterranean-style diet.

Although adequate hydration is considered essential for life, water intake is seldom considered along with nutrient intake [36]. However, the consumption of water can impact health and varies from one dietary pattern to another. Water consumption and adequate hydration status are usually associated with a MD. In contrast, in a Western style diet, sugary beverages are more likely to be consumed. Stookey et al., in response to an article on the possible inverse relationship between colon cancer risk and water intake [37], reported that an increase in water intake was associated with a reduced risk of breast cancer [38]. Although a small study, the results appear convincing and certainly warrant further research to confirm the findings.

### 3.1. Fatty Acids Commonly Consumed as Part of a Mediterranean-Style Diet

Dietary fatty acids in general are thought to be linked with cancer risk, development, and survival [39], despite some fats associated with a Mediterranean-style diet being regarded as beneficial in term of breast cancer outcomes [39]. The consumption of monounsaturated fatty acids (MUFAs) present in olive oil, have shown a benefit with regards to some types of breast cancer prevention and survival [40]. For instance, in 2012, Buckland et al. described the role of olive oil intake on breast cancer in postmenopausal women from Spain, Italy, and Greece, and identified no benefit in general [40]. However, there was an observed inverse association between olive oil intake and breast cancer in patients who had undergone Hormone Replacement Therapy (HRT), suggesting that olive oil might have a beneficial effect on oestrogen and progesterone receptor-negative breast cancer patients [40]. 

In contrast, a recent in vitro study revealed that the exposure of MCF-7 breast cancer cells (oestrogen and progesterone receptor positive) to phenolic extracts from Brava extra virgin olive oil showed a positive role in minimizing cell viability and promoting the production of ROS and the induction of cell death [41]. 

In addition to MUFAs, DHA, eicosapentaenoic acid (EPA), and other long-chain polyunsaturated fatty acids (PUFAs) are important fatty acids associated with a Mediterranean-style diet. The consumption of these fats has a promising effect on breast cancer prognosis. A cohort study performed on 3080 breast cancer survivors revealed a decrease in the mortality rate of those who consumed the highest quantity of DHA and EPA from foods [42]. No benefit was gained from DHA/EPA supplementation, and genotype was not assessed.

The role of fats commonly found in a Mediterranean-style diet can be demonstrated by investigating the effect of these fats on biomarkers of inflammation implicated in breast cancer development. Several studies have helped clarify this role, and these studies are presented in this review. The consumption of fish oil and some plant-based oils can play a protective role by reducing chronic inflammation and oxidative stress [42], both of which are risk factors for breast cancer. Omega-3 fatty acids play an important role in inhibiting NF-κB activity and pro-cytokine production, both of which are considered essential mechanisms of omega-3 fatty acid action in cancer, including cancer of the breast [43,44]. With regards to DHA consumption, high intake had an effect on altering the response of cancerous cells to the cytotoxic drugs used to treat breast cancer patients [45], which supports the value of these fatty acids in improving the outcomes of treatment in those with breast cancer. Rose et al. [46] investigated the role of EPA and DHA intakes, compared with linoleic acid, in inhibiting breast cancer development and metastasis. Mice that were fed a diet supplemented with DHA or EPA showed a reduction in the size of breast cancer tumours by inhibiting oxidative stress. A higher consumption of other omega-3 fatty acids (α-linolenic acid) led to a decreased risk of mammary gland cancers through the inhibition of NF-κB activity in mice [47]. 

Moreover, fatty acid intake may have an effect on cancer risk, initiation, and development through its role in the expression of genes (particularly oncogenes and tumour suppressor genes), and the mediation of cell signalling pathways [20,48]. This effect includes changes in enzyme expression, activity, and eicosanoid production [20,48]. For example, the protein levels of the tumour suppressor gene, *p53*, were decreased due to a high intake of linoleic acid, in a rat mammary tumour cell lines [21]. However, the expression of *p53* was up-regulated by DHA [20,21]. In addition, the exposure of breast cell lines to ω-3 PUFA (EPA and DHA) was related to the increase in the expression of two breast cancer suppressor genes, namely *BRCA1* and *BRCA2* [49]. In contrast, a reduction in the expression of *Her-2/neu*, an important oncogene in breast carcinoma aetiology and development, has been seen in BT-474 and SKBr-3 breast cancer cells after treatment with oleic acid supplement [50].

After finding a protective effect of marine fatty acid intake on breast cancer risk in postmenopausal women, Gago-Dominguez et al. investigated the relationship of their findings with the genotypes associated with glutathione-S-transferase (GST) enzyme activity [51]. They hypothesised that high-activity GST genotypes would remove lipid peroxidation products (that inhibit cancer cell proliferation) generated from marine ω3 fatty acids, leading to a greater risk of breast cancer. However, GSTM1 and GSTP1 were not associated with breast cancer risk, and the GSTT1 null genotype was associated with a 30% lower risk of breast cancer in postmenopausal Chinese women living in Singapore [51]. Postmenopausal women with GST polymorphisms leading to low or no GSTT1, GSTP1 and GSTM1 activity benefitted to a greater extent from the consumption of marine ω3 fatty acids in the context of breast cancer [51].

In the same cohort, the genotype of cyclin D1 (CCND1) was investigated for an association between genotype, breast cancer risk and interaction between risk and ω3 fatty acid consumption [52]. Although the CCND1 genotype was not associated with breast cancer risk, when analysed together with ω3 fatty acid consumptions, there was an association between risk, particularly advanced disease, and a high intake of ω6, a low intake of ω3, genotype (CCND1 GA ) or a complete lack of GSTT1 or GSTM1 activity [52].

In addition to the abovementioned genes, the arachidonyl lipoxygenase-12 (ALOX12) A835G genotype is associated with breast cancer risk [53]. The ALOX12 polymorphism A835G brings about a change in amino acid from glutamine to arginine at position 261, which brings about a change in ALOX12 expression, and this enzyme metabolises arachidonic acid [53]. The frequency of this polymorphism varies with ethnicity, and although breast cancer risk related to this genotype could well be modified by fatty acid intake, the interplay needs to be explored [17].

In general, it is premature to recommend the personalised consumption of olive oils and fish oils based on tumour type or genotype and, at this stage, it would be advisable to recommend the consumption of these oils to all women with breast cancer, or a predisposition to breast cancer.

### 3.2. Mediterranean Polyphenols 

Polyphenols have antioxidant activities and can be found in a wide variety of foods such as fruits, vegetables, cereals, chocolate, dry legumes, green tea, coffee, soy, and red wine [54]. There are four major types of polyphenols, including phenolic acids, stilbenes, flavonoids [54] and other polyphenols (Table 1).

The role of polyphenols in breast cancer has been widely investigated. Evidence revealed via several in vitro and in vivo observations will be explored below. 

#### 3.2.1. Epigallocatechin-3-Gallate (EGCG) 

Many observational studies have explained the important role of regular green tea intake on decreasing the risk of developing different types of cancer involving the breast [63,64]. Green tea contains the bioactive epigallocatechin-3-gallate (EGCG), a polyphenol which plays an effective role in preventing cancer development through multiple mechanisms. It acts as a methylation suppressor of tumour suppressor gene (TSG) promoter regions, an inhibitor of DNA methyltransferase (DNMT), and exhibits anti-cancer roles in both human and cancer cell line studies [65,66,67,68].

The treatment of MCF-7 breast cancer cell lines with EGCG prompted a reduction in cell proliferation and promoted apoptosis by reducing telomerase reverse transcriptase (hTERT) promoter methylation and suppressing histone H3 Lys9 acetylation. This induced a down regulation of hTERT expression [68] (which goes against the norm of increased promoter methylation increasing gene expression). Moreover, the mediated epigenetic induction of TIMP-3 levels has played a key role suppressing the invasiveness and gelatinolytic activity of matrix metalloproteinases-2 and 9 (MMP2 and MMP9) in breast cancer cells. Further, the MCF-7 cell line exposure to EGCG influenced the induction of tissue inhibitors for matrix metalloproteinases-3 (TIMP-3) as well as suppressing the activities of MMP2 and MMP9, which plays an important role in extracellular matrix degradation, cancer cell invasiveness, and metastasis [69]. 

Data from an animal study was used to elucidate the role of green tea polyphenols as well as black tea polyphenols in suppressing tumour growth in rats through a variety of mechanisms [70]. These include firstly a remarkable decrease in ROS production, secondly the blocking of NF-κB, Inducible Cyclooxygenase 2 (COX2) and Akt (protein kinase B) activities, and lastly overexpressing *p53* (a tumour-suppressor gene) [70]. Furthermore, Alotaibi et al. found that EGCG in two different forms (the bulk form and the polymer (poly[lactic-co-glycolic acid])-based nanoparticle (NP) form) induced a decrease in DNA damage in oxaliplatin-or satraplatin-treated lymphocytes obtained from colorectal cancer patients and healthy participants [71]. Also, low concentrations of EGCG were added into human peripheral leucocytes undergoing three phases that included stimulation with phytohemagglutinin, damage with genotoxic bleomycin, and incubation for sufficient time to allow DNA repair [72]. EGCG displayed a role in reducing bleomycin-induced breaks and endonuclease III sensitive sites, thus, suppressing the persistence of damage [72].

It is possible that EGCG may influence risk of breast cancer depending on the hormone receptor status of the tumour. In animal models, EGCG inhibited aromatase, which is involved in the conversion of androgen to oestrogen [73]. However, similar studies have not been reproduced in humans. In a prospective cohort study, Bao et al. partially supported these findings when following women over 9.1 years with triple-negative breast cancer [74]. The women who drank tea (89% of whom drank green tea) over the first 60 months following diagnosis had a decreased risk of breast cancer recurrence, and increased all-cause and breast cancer-specific survival than those who did not [74].

Given the mixed evidence of the benefit of EGCG on reducing breast cancer risk and recurrence, as well as the evidence garnered from in vitro and in vivo studies on gene expression associated with mechanisms of breast cancer, it is highly likely that there is an interplay between EGCG and genotype on breast cancer risk. However, to date, the evidence is lacking.

#### 3.2.2. Resveratrol 

Resveratrol is found in numerous plant-based products, including grapes, blueberries and some nut varieties. Also, it is abundant in red wine and dark chocolate [75], hence the focus on red wine intake. The epigenetic effects of resveratrol have been assessed in a number of studies. 

One of the abnormal events implicated in breast tumour development is silencing tumour suppressor genes through chromosome instability and DNA hypermethylation [76,77]. DNA methyltransferase 3b (DNMT3b) is an enzyme known to promote DNA hypermethylation [78]. Qin et al. found that a high concentration of resveratrol reduced the expression of DNMT3b in tumour cells, and elevated the expression in normal cells [79]. In addition, a number of miRNAs (miRNA21,–129, –204 and –489) were dysregulated in tumour cells in response to treatment with resveratrol [79]. Furthermore, 30 μM resveratrol treatment of breast cancer cell lines MCF7, MDA-MB 231, and HBL 100 for two days resulted in an observed surge in *BRCA1* and *BRCA2* mRNA. These genes are known as major breast cancer-promoting genes, but the protein expression of these genes remained constant [80]. Moreover, resveratrol displayed a cancer preventative role by recruiting DNMT1 to the *BRCA1* promoter and enhancing MCF-7 breast cancer cell silencing [81]. In addition, a combination of resveratrol and pterostilbene (a derivative of resveratrol) suppressed proliferation, induced apoptosis, down-regulated type III HDAC (histone deacetylase) (e.g., SIRT1), and affected DNA damage in MDA-MB-157 and HCC1806 breast cancer cells [82].

Resveratrol has been under investigation in term of its effect on inflammation and oxidative stress for some time. Treating female Sprague–Dawley rats with 100 µg of resveratrol led to an inhibition of 7, 12-Dimethylbenz(a) anthracene-induced tumorigenesis in rat mammary glands as well as suppressing subsequent effects including the activation of COX2, NF-κB and the expression of MMP-9. In the same article, the authors reported that the administration of resveratrol in MCF-7 breast cancer cells resulted in the suppression of NF-κB activity [83]. Additionally, Lio and colleagues have shown the effects of resveratrol in mitigating ROS generation and activating the antioxidant enzymes in fish that consumed 200 µg/g daily, compared with the control group who were not fed resveratrol at all [18]. 

In human trials, the role of circulating levels of resveratrol was evaluated in women at a high risk of developing breast cancer. The resveratrol-supplemented group revealed decreased Ras association domain family 1 isoform alpha (RASSF-1α) methylation, which is associated with enhancing cancer-promoting prostaglandin (PG)E_2_ [84]. 

Despite the fact that red wine contains beneficial polyphenols, including resveratrol, the effect of alcohol consumption on breast cancer risk is controversial. This controversy may be due to the fact that not all studies differentiate between the sources of alcohol intake, while others specify their interest in red wine intake only. In addition, accurate information on red wine intake over the life cycle is difficult to collect. Ethnicity, folate deficiency and genotype may all influence the impact of red wine intake and breast cancer susceptibility and recurrence, and not all researchers have taken this into consideration when designing their studies and analysing results.

Some studies have demonstrated the possible protective effects of red wine consumption in *BRCA1* mutation carriers [85,86], while, in general, moderate to heavy intake has been reported to have a detrimental effect, although a personalised approach is advised [87,88]. 

The benefit of red wine intake to some women with breast cancer was evident in observational studies and was attributed to the binding of resveratrol to the oestrogen receptor, which promotes *BRCA1* and *BRCA2* protein transcription [80,89]. Subsequent studies supported this finding with regards to breast cancer recurrence and mortality [80,89]. In contrast, there was no relationship observed between low and moderate alcohol consumption and breast cancer recurrence in a large cohort study where confounders, such as obesity and tobacco use, were corrected for [90].

In the context of cancers and alcohol consumption, it is important to consider folate blood concentrations. Although not proven in breast cancer, folate deficiency, together with high alcohol consumption is known to be a risk factor for colorectal, oesophageal and liver cancers together with genetic variants of the one-carbon metabolism pathway [91]. The genes involved include methylenetetrahydrofolate reductase (MTHFR). MTHFR C677T is linked with increased cancer risk in the context of low folate and methionine synthase (MTR) [91], and therefore a high folate intake would be recommended to women with this polymorphism.

Despite the evidence presented thus far, although the polyphenolic content of red wine could have a positive impact, the alcoholic content of red wine could have a negative impact on the epigenetic process with regards to breast cancer risk. A plausible mechanism of the effect of alcohol in epigenetic alteration is the increase in DNA methylation [87]. Meng and colleagues found a sustainable surge in E-cadherin as a likely promoter of methylation, and a decrease in p16 methylation amongst postmenopausal women [87]. This indicates that high alcohol intake before the age of 40 years may increase the risk of breast cancer after menopause. In addition, Purohit [88] demonstrated that moderate alcohol intake might play a role in increasing the oestradiol levels in postmenopausal women who undergo HRT. The increase of oestradiol levels can increase the risk of breast cancer recurrence [92], and therefore postmenopausal women on HRT should be deterred from alcohol consumption, and this includes red wine. 

In response to previous findings of the strong correlation between elevated insulin-like growth factor-I (IGF-I)/insulin resistance (IR) axis and increasing risk of breast cancer [93,94], a recent published study [95] examined the expression of 54 SNPs in IGF-I/insulin genes in response to alcohol intake and elevated BMI in 6567 women. A combination of three SNPs, including AKT1 rs2494740, AKT1 rs2494744, and AKT1 rs2498789, was found to significantly increase breast cancer risk in the context of high BMI and moderate and high alcohol intake (≥1 drink/day) [95]. This indicates how alcohol intake might be an important influential factor contributing to the occurrence of breast cancer in certain genotypes.

In conclusion, in addition to alcohol intake, nutrient intake may modify breast cancer risk. High folate intake is suggested to decrease the risk of breast cancer resulting from alcohol consumption in some genotypes [96]. The mechanism underlying this is the interaction between alcohol and folate-determining metabolism [97]. However, the role of alcohol consumption on breast cancer and its biomarkers is still controversial and requires additional investigation before personalised recommendations can be provided with confidence. In the meantime, it would be more appropriate to advise all with breast cancer, or at risk of breast cancer, to adhere to a low consumption of alcoholic beverages, and those with the risk genotype mentioned above and a high BMI, to abstain.

#### 3.2.3. Organosulfur Compounds 

These compounds include the sulforaphane group, which are dietary isothiocyanates that can be acquired from cruciferous vegetables such as sprouts and broccoli. The exposure of an oestrogen receptor-negative breast cancer cell line to a combination of sulforaphane, green tea polyphenols, and Tamoxifen, increased cell death and suppressed proliferation compared to Tamoxifen exposure alone [98]. This revealed the role of sulforaphane as elevating the cells’ sensitivity to Tamoxifen. In addition, sulforaphane plays an important role in suppressing HDAC in several cancer cells including breast, prostate, and colon [99,100,101]. 

As a source of sulforaphane, 200 g/day of broccoli protected both smokers and non-smokers against DNA damage [102], and smoking increases the risk of breast and some other cancers. Another major source of organosulfur compounds is garlic. Aged garlic extracts have been shown to increase cytokine release such as IL-2, IL-12 and tumour necrosis factor alpha (TNFα), reactivating lymphocyte proliferation, macrophage phagocytosis, the infiltration of lymphocyte and macrophage into cancer cells, and stimulating NF-κB activity [103,104,105,106].

Although data regarding gene–nutrient interventions is not readily available, women at risk of DNA damage from smoking or with a low antioxidant capacity (due to genotype) would be encouraged to consume diets high in organosulfur compounds.

#### 3.2.4. Quercetin 

This flavonoid compound is found in fruits, such as apples and grapes, and in specific vegetables such as onions, kale, broccoli, lettuce, and tomatoes. Quercetin has been shown to have a protective effect on anti-cancer activity [107] by suppressing the activity of COX2 [108], and NF-κB [109]. In an in vitro study, using MCF-7 breast cancer cells, quercetin suppressed Twist via the p38 MAPK pathway [110], and downregulated Bcl2 and upregulated Bax [111], and thereby induced apoptosis. Roslan et al. have found that quercetin is a beneficial nutrient affecting inflammation, oxidative stress, and apoptosis pathways in diabetic mice [112]. Thus, these beneficial influences on breast cancer biomarkers might play a role in inhibiting disease development and recurrence. However, the bioavailability of quercetin is poor. For this reason, Niazvand et al. (2019) et al. investigated the use of solid lipid nanoparticles for the delivery of quercetin [113]. They found it to be more toxic to MCF7 cells, than free quercetin, with a markedly lower half maximum inhibitory concentration (IC50) [113]. The use of the slow release of quercetin from nanoparticles is yet to be applied in breast cancer patients.

More and more research is being carried out on the synergistic effects of quercetin and anticancer drugs, for example Doxorubicin, which is a drug used to treat a number of cancer types, including breast cancer, shows enhanced activity when administered with quercetin in in vitro assays [114]. It is also anticipated that quercetin may decrease the toxic effects of Doxorubicin, as the combination was well tolerated by non-cancer breast and myocardial cells [114]. It remains to be seen as to whether thi genotype will have an impact in this context. 

#### 3.2.5. Kaempferol 

Kaempferol is a phytoestrogen and a flavonoid that is found in a variety of fruits and vegetables, for example in grapefruit and grapes, as well as broccoli and beans. Kowalski et al. have shown that the treatment of J774.2 macrophages with kaempferol, in combination with apigenin and resveratrol, plays a significant role in suppressing the expression of TNF-α and interleukin-1β (IL-1β). Treatment with kaempferol also blocked the transcription of these two cytokines by reducing the level of mRNA [115]. Additionally, kaempferol was administrated in human embryonic kidney (HEK)293 cells with various effects including the reduction of TNF-induced IL-8 activity, the genetic expression of IL-8, blocking NF-κB activity, and lowering ROS production [116], and this may be useful to reduce inflammation and thereby lower susceptibility to breast cancer.

Kaempferol inhibits the progression of a number of cancer types and in breast cancer, it is believed to inhibit cancer cell proliferation via cell cycle arrest and the upregulation of *p53* [117], and also acts by blocking cell invasion [118]. In triple-negative breast cancer cells, a low dose of kaempferol (20 µmol/L) inhibited the migration of cells, but in ER+/PR+, and HER2+ cell lines, a much higher dose of kaempferol was required (200 µmol/L) [118]. The mechanism of antiproliferative activity in MCF7 cells, an ER+/PR+ cell line, was shown to occur via the inhibition of glucose and lactic acid uptake [119], and this may also hold true for all breast cancer genotypes. When compared with genistein and resveratrol amongst other bioactive compounds, kaempferol was the most potent inhibitor of cell proliferation [119].

Similar to the situation with quercetin, the bioavailability of kaempferol needs to be enhanced, and synergistic effects need to be elucidated. 

#### 3.2.6. Apigenin 

Plant-based foods such as parsley, chamomile tea, oranges, and grapefruit are sources of this flavonoid compound. Treating breast cancer cells with apigenin played a role in inhibiting cell proliferation, promoting cell cycle arrest and ROS generation [120]. This polyphenolic compound has anti-inflammatory effects as demonstrated by its inhibitory effect against the action of 5-lipoxygenase and cyclooxygenase, in particular COX2, which plays a role in the synthesis of inflammatory pathway mediators [121]. A recent published abstract outlined the role of apigenin in overcoming drug resistance in chemotherapy [59]. Apigenin successfully caused a reduction in the mRNA expressions of multi-drug resistance 1 (MDR1). Also, it showed a notable impact in the down-regulation of P-glycoprotein (P-gp). P-gp overexpression is one of the important causes of drug resistance [59]. 

### 3.3. Micronutrients Commonly Found in a Mediterranean-Style Diet

#### 3.3.1. Zinc

Zinc contributes to the regulation of many intra- and extra-cellular pathways involved in cell proliferation, differentiation, development, apoptosis, and transformation [122,123,124]. Zinc is found in abundance in egg yolks, meat, poultry, legumes, peanuts, non-fat milk powder, cheddar cheese, wheat, and cocoa [125]. It has been proposed that zinc intake might be essential to protect against breast cancer progression and recurrence [126]. According to a recent study, zinc deficiency promoted the abnormal growth of MCF-7 breast cancer cells, and cell exposure to zinc supplements increased the expression of two important genes namely *CDKN2A* and *p53*, and decreased the expression of mdm2 [126]. *CDKN2A* and *p53* are important genes for inducing apoptosis and promoting survival, whereas mdm2 acts as a promoter for *p53* gene degradation [127].

The deficiency of this micronutrient plays an important role in DNA replication and repair by disrupting proteins and molecular actions involved in this process [123,124]. Zinc-deficient rat glioma C6 cells exhibited a surge in oxidative stress and DNA single-strand damage, as well as an impairment in DNA-binding to NF-κB and activator protein 1 (AP1) [128]. In addition to this indicative data from in vitro studies, data from in vivo studies suggested the protective impact of zinc supplement from breast cancer [15,129,130]. A Canadian study suggested that a 10-year intake of a zinc supplement played a role in preventing breast cancer development among premenopausal women. Similarly, this supplement showed a positive influence among postmenopausal women when consumed with multiple vitamins such as beta-carotene, vitamin C and vitamin E [129]. 

Although there is limited evidence on the direct effect of zinc intake on breast cancer, in some studies, the effect of zinc intake has been explored. For instance, Kelleher et al., 2009 [130] examined the effect of zinc deficiency among nursing women during lactation. Zinc deficiency was implicated in breast cancer progression and development as it resulted in improper zinc metabolism and therefore, breast cancer [130]. Furthermore, zinc is considered as one of the factors contributing to genetic abnormalities thus leading to DNA instability and a greater risk of breast cancer development. For example, serum levels of zinc were examined among *BRCA1* carriers and non-carriers and a positive impact was found such that both groups with high serum levels of zinc had a lower incidence of breast cancer [15]. The protective impact observed was statistically significant among *BRCA1* gene carriers [15]. More clinical trials are required to confirm these effects.

#### 3.3.2. Selenium (Se)

Se is a micronutrient essential for human health and associated with multiple diseases including cancers [131]. Se is naturally obtained from food such as onions, broccoli, and grains, depending on the Se content of the soil upon which they were grown. There is some controversy regarding supplementation with Se, in part due to variations in study design wherein different forms of Se were used, and in the enrolment of populations with sufficient serum Se [132].

In vitro studies were used to evaluate the effects of Se-yeast and methylseleninic acid intake on oxidative stress, growth inhibitory activity and apoptosis in ER-positive MCF-7 and triple-negative MDA-MB-231 cells [133]. Lower levels of ROS were produced in response to treatment with Se-yeast to induce early apoptosis [133]. Also, there was a modest effect of Se-yeast on growth-inhibitory activity. In contrast, methylseleninic acid promoted high levels of ROS production and high proliferation inhibitory activities. However, it also induced late apoptosis [133]. This indicates the significance of the form of selenium used [134,135]. In addition to studies on different types of Se, Se was found to exert a protective effect by reducing the oxidative stress induced by mobile phone-induced electromagnetic radiation in breast cancer cells [136]. A recent human trial revealed evidence on the linkage between selenium serum level and breast cancer recurrence [137]. The trial examined serum level of selenium among 546 women diagnosed with invasive breast cancer, for a 3.8 year period [137]. Those with >81.0 µg/L selenium level had 82.5 percent overall survival, i.e., higher rates than those with a low serum level of selenium [137].

In conclusion, an adequate intake of zinc is essential, as zinc deficiency can impair DNA replication and promote abnormal cell growth. On the other hand, adequate levels can lower the risk of breast cancer in *BRCA1* gene carriers. Adequate serum Se concentrations have also been shown to be beneficial. However, both high and low levels may be harmful. To date, there is no evidence to support the personalisation of Se based on genotype.

## 4. Limitations

In multiple studies, the focus of dietary impact was on breast cancer outcomes. However, strong evidence to support the benefit of various dietary patterns is still lacking [33]. One possible reason for this is that breast cancer patients and survivors might change their eating patterns after diagnosis. For example, the Women’s Healthy Eating and Living (WHEL) trial evaluated the dietary intake of 3109 women before and after breast cancer diagnosis [138]. There was a notable increase in fruit, vegetable, and fibre intake and a decrease in high-fat dietary consumption involving fast food, after diagnosis [138]. Another possible reason for the lack of strong evidence is the change in diet over the life cycle. This change includes the effect of maternal diet during the early embryonic period, gestation and lactation, and maternal diet during these time periods may influence breast cancer risk and development of the offspring later in life. This influence is demonstrated by the consumption of saturated fatty acids during gestation and/or lactation, and the reported increase of breast cancer in adulthood [139]. These fats do not form part of a Mediterranean-style dietary pattern and are more consistent with a Western style diet. Dietary exposure during foetal development is not the focus of this review. However, the authors refer the reader to a couple of informative articles focused on this topic [140,141].

A common means of assessing adherence to a Mediterranean-style dietary pattern is via the calculation of a dietary adherence score. No single scoring system has been used across all relevant studies, and hence variations in scoring, and therefore correlations between scores and biomarkers of disease risk and molecular pathways, may be inconsistent.

A Mediterranean-style dietary pattern, from a medical/health research perspective, is often assessed on dietary intake only. However, such a dietary pattern incorporates a particular lifestyle that includes physical activity, culinary activities that involve friends and family, and conviviality (i.e., the value of the meal is not just nutritional, but social and cultural) [142]. Generally, in research, we do not take these non-nutritional components into account.

Adherence to a Mediterranean-style dietary pattern is often assessed through the application of scoring system. These scoring systems differ, and therefore results/correlations may not be directly comparable. In addition, a high score on a MD adherence questionnaire does not necessarily imply adequate nutrient intake [143], although this is how the score is sometimes interpreted. 

A Mediterranean-style dietary pattern constitutes a moderate intake of alcohol [144], but the type of alcohol consumed is not always defined in the questionnaires used. In a MD diet, this intake is characterised by multiple conditions including moderate intake, a preference for red wine and consumption with meals [145,146,147]. Although a benefit was observed in some studies, there were others, which have been discussed herein, that reported an increased breast cancer risk with alcohol intake. However, this increased risk was in combination with other factors such as a high BMI [95], the use of HRT [88], and a high intake of alcohol before menopause [87]. In order to draw conclusions regarding the benefit of red wine consumption, information on alcohol intake needs to be detailed so that confounding factors can be corrected for and overall alcohol consumption can be taken into account.

## 5. Conclusions

From the numerous studies presented in this review, it can be clearly seen that following a MD diet could be beneficial to health in general and help protect against breast cancer risk and recurrence in particular. To further support this notion, nutrients contained in a typical MD diet displayed a positive impact on biomarkers of inflammation, DNA damage, oxidative stress, and genetic alterations, all of which may influence breast cancer outcomes.

Clearly, the host genotype also plays a role, not only in susceptibility to breast cancer, but also with regards to benefits obtained from typical foods and nutrients that make up the MD diet. In order to maximize the effect of nutrients on genotype, further research is required to discover additional diet–gene interactions. In addition, it is necessary to define the concentrations of nutrients, and synergistic effects between nutrients, as well as between nutrients and anticancer drugs in relation to genotype.

## Figures and Tables

**Table 1:** Classification of polyphenols commonly found in a Mediterranean-style diet.

Polyphenol	Sub-Groups	Food Source
Phenolic acid	Benzoic acid Cinnamic acid	Mostly in grains [55,56,57].
Flavonoids	Anthocyanins Flavan-3-ols Flavones Flavanones and Flavonols	Vegetables, fruits, seeds, some cereals, together with wine, tea and certain spices [58].
Flavonoids (Flavones)	Apigenin	Parsley, grapefruit, chamomile tea, oranges, and grapefruit [59].
Flavonoids (Flavonols)	Quercetin Kaempferol	Onions, broccoli, apples and berries [60].
Flavonoids (Flavanols)	Catechin Epicatechin Epigallocatechin Epigallocatechin gallate	Many types of fruits, red wine, green and red tea [61].
Polyphenolic amides	Capsaicinoids and avenanthramdes Resveratrol (a stilbene)	Chilli peppers and oats (respectively) grapes and red wine [62].
Other Polyphenols	Ellagic acid and gallic acid	Berry fruits, e.g., strawberries and raspberries, and in the skins of different tree nuts [62].
